# Delayed Diagnosis of Spinal Dural Arteriovenous Fistula: A Case Report and Scoping Review

**DOI:** 10.3390/jcm13030711

**Published:** 2024-01-25

**Authors:** Tatsuya Tanaka, Fumitaka Yamane, Ryohei Sashida, Yu Hirokawa, Tomihiro Wakamiya, Yuhei Michiwaki, Kazuaki Shimoji, Eiichi Suehiro, Keisuke Onoda, Akira Matsuno, Tadatsugu Morimoto

**Affiliations:** 1 Department of Neurosurgery, International University of Health and Welfare, Narita Hospital, 852 Hatakeda, Narita 2868520, Japan; 2 Department of Orthopedic Surgery, Faculty of Medicine, Saga University, Saga 849-8501, Japan

**Keywords:** differential diagnosis neuroimaging findings, misdiagnosis, myelitis, spinal arteriovenous fistula, spinal arteriovenous malformation, spinal vascular disease, clinical presentation

## Abstract

Spinal dural arteriovenous fistula (SDAVF) is among the most common arterial shunt diseases typically found in middle aged or older men. Herein, we aimed to clarify the reasons for misdiagnoses and delayed diagnoses of SDAVF, determine how these affect prognoses, and establish how they can be prevented. We conducted a PubMed/MEDLINE literature search using “spinal dural arteriovenous fistula”, “delayed diagnosis”, “late diagnosis”, and “misdiagnosis” terms. We identified 18 articles, including 965 SDAVF cases. Patients were predominantly males (71.8–100.0%) (mean age: 53.5–71.0 years). Misdiagnoses rates varied (17.5–100.0%) and encompassed many conditions. The mean time between early manifestations and confirmed diagnosis was approximately 10–15 months and from the first radiologic image revealing dural arteriovenous fistula (DAVF) features to diagnosis was 9.2–20.7 months. Posttreatment outcomes showed a significant improvement in motor functions, gait, and micturition, particularly in patients exhibiting preoperative symptoms over a short period. SDAVF is frequently misdiagnosed or subject to delayed diagnosis, causing poor clinical outcomes. SDAVF symptoms including progressive lower-limb weakness, paresthesia, and vesicorectal dysfunction are indications for spinal magnetic resonance imaging with subsequent spinal angiography, wherein DAVF is evidenced by extensive T2 hyperintensity and flow-void abnormalities. We reported a representative case with delayed diagnosis.

## 1. Scoping Review Introduction

Spinal dural arteriovenous fistula (SDAVF) is among the most common spinal arteriovenous shunt diseases, including SDAVF, intramedullary arteriovenous malformation (AVM), peripheral arteriovenous fistula (AVF), and perimedullary AVF. It is typically found in middle-age or older males’ thoracic spines. The disease manifests as a fistula between the radiculomeningeal artery and the radiculomedullary vein in the dura mater, near the spinal nerve root. This causes high-pressure blood flow from the artery to affect the veins around the spinal cord, causing their expansion and subsequent venous congestion and promoting myelopathy that leads to neurologic symptoms, such as numbness in the lower extremities, gait disturbance, and urinary retention.

SDAVF progression may last from several months to years, resulting in irreversible spinal cord damage. However, if it is diagnosed early and the fistula is closed, disease progression can be halted, and complete recovery is likely [1]. However, the condition is easily misdiagnosed, and it can take some time to arrive at a definitive diagnosis [2]. In a previous study, the median time from onset to treatment was longer in misdiagnosed patients than in correctly diagnosed ones (11 vs. 4 months). Misdiagnosed patients developed additional disabilities by the time a correct diagnosis was made (Aminoff–Logue gait grade of 3.6 vs. 2.1) and thus achieved significantly smaller improvements after the treatment (Aminoff–Logue gait grade of 3.0 vs. 1.1) compared to those who were correctly diagnosed with spinal DAVFs [1].

In this scoping review, we summarize the typical features of SVDAF. Through a literature review, we aimed to determine the reasons for diagnostic delay and misdiagnoses of SVADF, elucidate the relationship between diagnostic delay and prognosis, and identify the factors that facilitate early diagnosis. Moreover, we reported a representative case in which diagnosis was delayed.

## 2. Scoping Review

For this scoping review, we searched for articles reporting delayed diagnosis and outcomes of thoracic spine SDAVF. Our literature search was conducted on the PubMed/MEDLINE database (NCBI-National Center of Biotechnology Information, NIH, US Government). The search included all articles written in English and published before August 2023. The search words were “spinal dural arteriovenous fistula”, AND “delayed diagnosis”, OR “late diagnosis”, OR “misdiagnosis”.

The inclusion criteria were as follows:

- Prospective or retrospective cohort studies, case–control studies, and case series.
- Patients of both sexes aged 19 years or older.
- SDAVF of thoracic spine (with the arterial blood supply at the thoracic level), including idiopathic, iatrogenic, traumatic, and syndromic types.
- Patients treated with open and endovascular procedures were included, as were those who underwent repeated surgery.
- The exclusion criteria were as follows:
- Reports not including SDAVF cases of the thoracic spine.
- Spinal arteriovenous shunt diseases other than SDAVF (intramedullary AVM, peripheral AVF, and perimedullary AVF) were excluded from each article.
- Inadequate reporting of demographic characteristics, imaging features, or intraoperative procedures.

**Figure 1:**
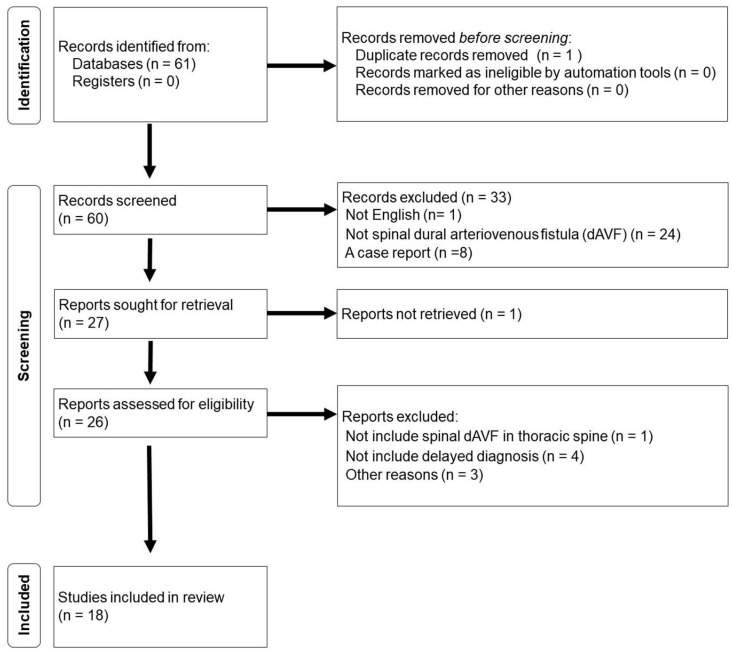
Identification of thoracic spinal dural arteriovenous fistula studies from database and register.

The scoping review methodology is shown in Figure 1. Overall, 61 studies were examined, 18 of which were included in our review. We executed rigorous data collection and introduce this in Table 1.

## 3. Results

The number of patients with dural arteriovenous fistula (DAVF) in the 18 articles reviewed ranged from 7 to 326 per study. There were 965 cases in the reviewed papers. Of these, SDAVF location was clearly defined in 825. SDAVF was thoracic in 561 of these 825 cases (68%) [1,2,3,5,6,7,8,10,11,12,13,14,15,16,17,18]. Most of the included cases were male (71.8–100%) [1,2,3,4,5,6,7,8,9,10,11,12,13,14,15,16,17,18]. The mean patient age ranged from 53.5 to 71.0 years [1,2,3,4,5,6,7,8,9,10,11,12,13,14,15,16,17,18].

The misdiagnosis rate was 17.5–100.0% [1,2,3,5,6,7,8,9,12,16,18]. The conditions where SDAVF was misdiagnosed included spondylosis/spinal degenerative disease, tumor, demyelination, inflammatory myelitis, myelopathy, peripheral neuropathy, spinal-cord infarction, inflammatory disease, syrinx, benign prostatic hyperplasia, neuromyelitis optica, multiple sclerosis, Guillain–Barré syndrome, polyneuritis, peripheral vascular disease, POEMS (polyneuropathy, organomegaly, endocrinopathy, monoclonal protein, skin changes) syndrome, post-polio syndrome and syringomyelia, refractory restless-legs syndrome, mixed headache, and unexplained spinal cord lesions [1,2,3,5,12,13,16,18].

The mean duration from onset of symptoms to diagnosis was 10.15 months (median, 10–24 months; range, 1–120 months) [3,4,6,8,10,11,12,15,16,17]. The mean time from the first radiologic findings of DAVF to confirmed diagnosis was 9.2–20.7 months (range, 1–168 months) [2,5,9]. The mean time from symptom onset to treatment initiation in the entire sample was 12–39 months (range, 7–144 months) [1,3,8,13,14]. The median time from symptom onset to diagnosis was 41.5 months in their misdiagnosis group and 5 months in their nonmisdiagnosis group [6]. Another report found median times from symptom onset to diagnosis of 2.3 years in their misdiagnosis group and 0.9 years in their nonmisdiagnosis group [3]. Another study found the time from initial magnetic resonance imaging (MRI) to diagnosis to be significantly long in misdiagnosed patients assessed for differential diagnosis (281 vs. 22 days) [5].

Post-treatment outcomes were reported in 16 of the studies. These were measured by Aminoff–Logue scores in 13 [1,2,3,4,6,7,8,9,10,11,12,13,14,16,17,18], manual muscle testing in 1 [16], the modified Rankin Scale in 1 [10], and the American Spinal Injury Association Impairment Scale in 1 study [14]. The posttreatment increases in the Aminoff–Logue score ranged from 42.3 to 85.0% overall [6,7,8,12,13], from 32.0 to 59.5% for gait [2,3,4,6,9,10,11,13,14,16,17,18], and from 0 to 35% for micturition [2,3,4,6,13].

Variables that were assessed for their relationship with clinical outcomes included the duration between symptom onset and intervention, the severity of the initial deficit, the extent of cord edema, treatment success or failure, and the presence or absence of residual fistula [8]. A noteworthy effect was produced by preoperative symptom duration, with short duration linked to improved motor (median, 0.8 vs. 3.1 years; *p* = 0.001) and urinary functions (median, 0.8 vs. 2.2 years; *p* = 0.040) postoperatively [3].

### Case Description

**Figure 2:**
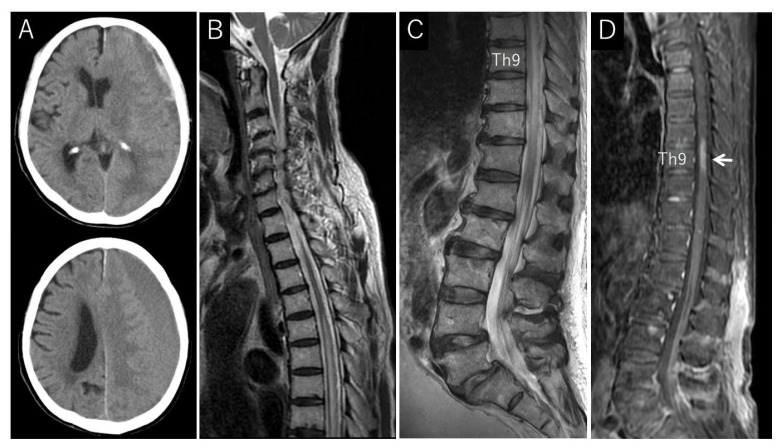
Computerized tomography (CT) and magnetic resonance (MR) images from a patient with dural arteriovenous fistula (DAVF); (**A**) CT scan of the head showing left chronic subdural hematoma; (**B**) sagittal T2-weighted MR image of cervicothoracic spine showing cervical canal stenosis, spinal cord edema, and flow voids around the cord; (**C**) sagittal T2-weighted MR image of thoracolumbar spine, highlighting spinal cord edema and flow voids around the cord; (**D**) sagittal contrast-enhanced MR image displaying enhancement of spinal cord condition (arrow).

An 84-year-old man with a history of chronic subdural hematoma and cervical canal stenosis was referred to our neurology department, presenting progressive weakness in both lower extremities, sensory disturbances, and bladder–rectal dysfunction. His symptoms began 8 months earlier (Figure 2A,B).

During his physical examination, the patient was awake, alert, and oriented, with stable vital signs. A neurological examination revealed significantly decreased muscle strength and sensation in his lower extremities. At 7 months post-symptom onset (before the patient’s referral to our department), an initial lumbar spine MRI examination showed an abnormal thoracic spinal cord extension at T6–T11, with increased fluid signal within these parts of the cord (Figure 2B,C). The application of contrast showed enhancement at Th8–9 (Figure 2D).

**Figure 3:**
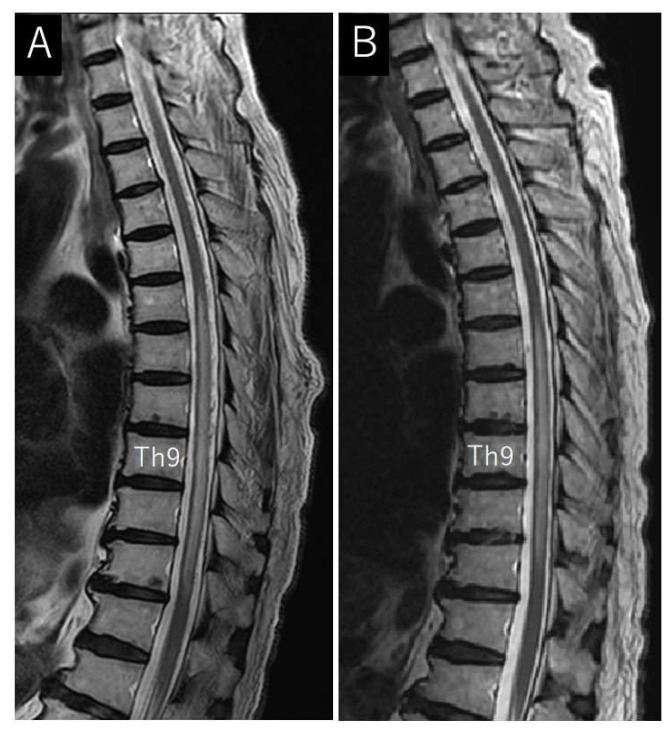
Pre- and post-treatment magnetic resonance (MR) images from a patient with dural arteriovenous fistula (DAVF); (**A**) pretreatment sagittal T2-weighted MR image of the thoracolumbar spine showing spinal cord edema and flow voids around the cord; (**B**) posttreatment sagittal T2-weighted MR image of the thoracolumbar spine showing edema reduction and flow-void disappearance.

**Figure 4:**
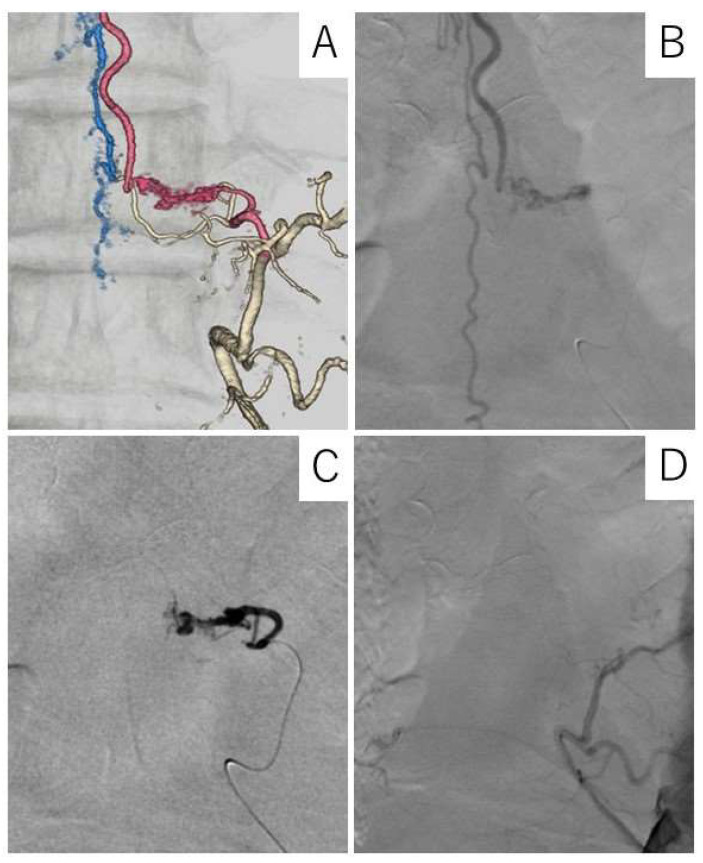
Three-dimensional digital subtraction angiographic (DSA) images and spinal X-ray of a patient with dural arteriovenous fistula (DAVF). (**A**) Anteroposterior DSA view of the right Th-9 and -10 segmental arteries; DAVF, intradural arterialized vein, and dilated perimedullary veins can be seen; (**B**) anteroposterior DSA view of right Th-9 segmental artery displaying a shunt lesion from selective catheterization of right Th-9 radicular branch and a tangle of arterialized veins in the central spinal canal; fistula can be seen at right Th-9 nerve sleeve; (**C**) post-treatment X-ray showing the presence of N-butyl cyanoacrylate (NBCA); (**D**) post-treatment anteroposterior DSA view of right Th-9 and -10 segmental arteries showing disappearance of the spinal DAVF.

We conducted a second MRI 8 months post-symptoms onset, showing edema at levels T6–T11, as well as numerous abnormally dilated vascular flow voids around the spinal cord, consistent with SDAVF (Figure 3A). Under local anesthesia, a right-groin common femoral artery puncture was performed for access, and selective spinal angiography was performed with a 4-French Shepherd hook catheter. We identified a dural fistula supplied by left Th9 radicular artery connected to left Th9 radicular veins (Figure 4A). Our team of neurologists and neurosurgeons discussed the diagnostic features, and embolization was selected as the treatment method.

Under general anesthesia, a right-groin common femoral artery was punctured with a 5-French sheath, and super-selective angiography and endovascular embolization with n-butyl-2-cyanoacrylate (NBCA) of the left Th9 radicular artery were implemented. Complete obliteration was achieved and verified on angiogram (Figure 4B–D).

The patient exhibited immediate improvement in his sensory function, with progressively great ameliorations detected on each postoperative recovery day. The postoperative MRI showed a slight decrease in spinal cord edema and no abnormally dilated vascular flow voids around the spinal cord (Figure 3B). The patient was discharged.

At the patient’s 6-month posttreatment follow-up, he reported continued leg weakness, paresthesia, and bladder–rectal dysfunction.

## 4. Discussion

We investigated the factors that can interfere with early SDAVF diagnosis and suggested measures to mitigate them. We found the main cause of delayed or incorrect diagnosis to be the failure to recognize the characteristics of these lesions, clinically and in neuroimaging findings.

### 4.1. Symptoms

A typical patient with SDAVF has symptoms similar to thoracic myelopathy, epiconus syndrome, and conus medullaris syndrome [1]. Thoracic myelopathy produces motor weakness in the proximal lower extremities, posterior funiculus dysfunction, and amplified deep-tendon reflexes. Epiconus syndrome is characterized by progressive numbness, developing upward from the distal lower extremities. In conus medullaris syndrome, bowel and bladder dysfunction and claudication can occur while walking, bathing, drinking, sleeping, gardening, and even singing [1,19]. Additionally, these symptoms are seen in SDAVFs and are significantly different from spinal canal stenosis [1,19]. If a patient’s symptoms do not fit with common spinal stenosis, SDAVF should be included in the differential diagnosis.

### 4.2. Neuroimaging Findings

MRI can detect the venous congestion of the spinal cord characteristic of SDAVF with up to 100% sensitivity [20]. Most patients with SDAVFs present high peripheral signal intensity on T2-weighted MRI [21]. Venous congestion spreads from lower thoracic spinal cord to conus medullaris, regardless of the location of the fistulas, potentially at a different level [22].

A peculiar finding in SDAVF patients is enlarged subarachnoid spinal cord veins, enabling the differentiation of SDAVF from other diseases. The specificity reported for high-signal intensity on T2-weighted MRI and flow voids is 97% [20,23]. Although venous congestion can suggest intramedullary tumors, myelitis, syringomyelia, or demyelinated disease of the spine, the additional presence of dilated veins is a reliable indicator of SDAVF [24,25]. T2-weighted MRI enables the visualization of these abnormal structures as flow voids around the spinal cord. However, not all cases display flow voids on MRI. Multidetector computed tomography angiography (MDCTA) and enhanced magnetic resonance angiography (MRA) with gadolinium can be useful additional tools in the detection of abnormal vessels around the spinal cord [26,27]. Even with lumbar MRI, venous congestion and flow voids might be found at the conus medullaris [28]. If lumbar MRI shows conus medullaris lesions, thoracic MRI should be performed to identify venous congestion of the spinal cord and vascular flow voids around the cord.

Furthermore, MDCTA and enhanced MRA are useful as preoperative tests. The positional relationship between the vertebral body and abnormal blood vessels is easy to understand, and the level of the fistula can be predicted to some extent, making it possible to reduce the amount of contrast agent and radiation exposure during digital subtraction angiography (DSA) [26,27]. Performing MDCTA or enhanced MRA before DSA is useful; however, this test cannot replace DSA at this time, and DSA is ultimately required.

Having knowledge of the anatomy of the arteries that supply spinal arteries is crucial during DSA. In the thoracic and lumbar regions, intercostal and lumbar arteries serve as segmental arteries that supply spinal arteries. About 1–2% of cervical SDAVFs are located at the craniocervical junction, of which 12% are caused by arterial feeders from the external carotid artery [29]. A correct diagnosis can be challenging, and a high rate of initial misdiagnosis is possible. DSA should always include the internal carotid, external carotid, and vertebrobasilar arteries.

### 4.3. Misdiagnosed Diseases

The primary reason for delayed and incorrect SDAVF diagnosis is that SDAVF is easily misrepresented as other diseases. Spinal degenerative diseases and myelitis are particularly common misdiagnoses.

#### 4.3.1. Spinal Degenerative Disease

The initial symptoms of SDAVF are nonspecific motor and sensory deficits affecting the lower extremities. Symptoms worsen and improve while walking and resting. These symptoms are similar to intermittent cauda equina claudication seen in lumbar spinal stenosis. First findings are often obtained by lumbar MRI. If there is evidence of spinal stenosis, the latter might be misdiagnosed as the cause. To avoid this, considering whether all of the symptoms can be explained by lumbar spinal canal stenosis is crucial.

SDAVF can be worsened by walking, singing, Valsalva maneuver, and drinking alcohol [1,19]. Considering difficult-to-explain symptoms because lumbar spinal lesions can also produce weakness of the iliopsoas muscle, sensory disturbance below the groin, and the Babinski sign is essential. SDAVF should be considered if there is a slow progression of symptoms. Particular attention should be paid to abnormalities at the thoracolumbar junction (intramedullary high-signal intensity, abnormal vascular images in the subarachnoid space) on lumbar spine MRI.

#### 4.3.2. Myelitis

SDAVF is easily mistaken for myelitis due to the long intramedullary hyperintensities seen on T2-weighted MRI images and abnormal vascular images in the subarachnoid space that characterize both conditions. While the course of SDAVF is often slow, it can worsen rapidly with exercise or other activities. Myelitis has an acute onset [30]. SDAVF often has a mild increase in the number of cells in the cerebrospinal fluid.

Steroid administration reportedly causes acute clinical deterioration in SDAVF patients. When a patient with suspected myelitis shows worsening symptoms with steroid administration, differential diagnostics for SDAVF should be carried out [23,31].

### 4.4. Diagnostic Procedure

**Figure 5:**
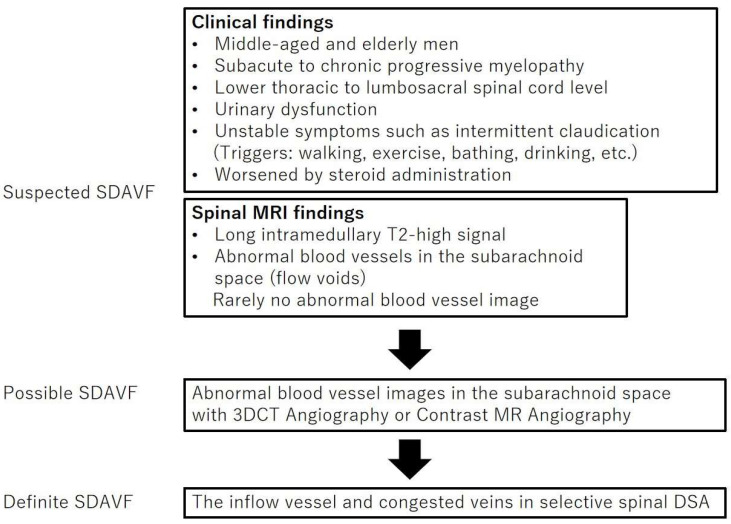
Diagnostic process for spinal dural arteriovenous fistula detection. SDAVF represents spinal dural arteriovenous fistula; MRI, magnetic resonance imaging; 3DCT, three-dimensional computed tomography; DSA, digital subtraction angiography.

Figure 5 provides a flow diagram for the diagnostic process in SDAVF. SDAVF may first be suspected based on clinical and spinal MRI findings.

The study patients mostly comprised middle-aged or elderly men. The reason for this demographic feature is unclear, but differences in sex hormones might increase the likelihood of SDAVF in middle-aged men compared to women [8]. Arteriovenous fistulas in female mice have reduced the patency, velocity, and magnitude of shear stress and laminar flow during fistula remodeling [32]. There is sex-specific differential expression of proteins involved in thrombosis, response to laminar flow, inflammation, and proliferation [32]. Arteriovenous fistulae (AVF) fail to mature more frequently in female patients and might be compared with male patients. Thus, SDAVF should be suspected in middle-aged or elderly men.

Minimally invasive 3D computerized tomography (CT) or contrast-enhanced MRA are then used to detect abnormal blood vessels. Finally, selective spinal angiography is used to make a definitive SDAVF diagnosis.

### 4.5. Treatment and Outcomes

SDAVF treatment includes microsurgery and embolization. The treatment goal is to prevent the flow of blood from the proximal intradural vein and fistula. If treatment is delayed, lower-limb weakness, paresthesia, and bladder–rectal dysfunction can persist. Early diagnosis and treatment are therefore essential; results emphasize their importance in SDAVF.

## 5. Conclusions

SDAVF is frequently misdiagnosed due to its nonspecific features, and late diagnosis can worsen the prognosis. Spinal MRI should be conducted in patients presenting symptoms such as slowly progressive lower-limb weakness, paresthesia, and vesicorectal dysfunction. Spinal MRI findings such as extensive T2 hyperintensity and flow-void abnormalities should raise the suspicion of DAVF. And spinal angiography should be ordered. Delayed diagnosis of DAVF usually causes long-term irreversible neurological complications. Thus, early diagnosis and intervention are crucial.

## Figures and Tables

**Table 1:** Summary of studies assessing misdiagnoses and delayed diagnoses of spinal dural arteriovenous fistula.

Author and the Year	Study Population (n = Number of Patients)	Patient Age, Mean, Median (Range)	Males, n (%)	Spinal dAVF in Thoracic Spine, n (%)	Misdiagnosis, n (%)	Misdiagnosed Disease, n	Duration from Symptom Onset to Diagnosis	Duration from the First Radiologic Imaging Conduction That Showing Features of dAVF to Diagnosis	Duration from Symptom Onset to Treatment	Outcome Evaluation Method	Outcome of All	Outcome of Gait	Outcome of Micturition
Ronald AA et al., 2020 [3]	46	median 63 (57–71)	35 (76.0)	28 (60.9)	16 (34.8)	spondylosis/degenerative disease 6, idiopathic autoimmune inflammatory myelitis 2, Guillain-Barré syndrome 1, disc herniation 1, peripheral vascular disease 1, tumor 1, multiple sclerosis 1, neuromyelitis optica 1, POEMS (polyneuropathy, organomegaly, endocrinopathy, monoclonal protein, skin changes) syndrome 1,peripheral neuropathy 1	median 2.3 (misdiagnosis) vs. 0.9 years (no-misdiagnosis)	NA	median 1.2 years	Aminoff-Logue Score	NA	improved 57% stable 30% worsened 14%	improved 35% stable 54% worsened 11%
Zhang L et al., 2020 [2]	65	mean 53.5 (25–81)	52 (80.0)	45 (66.2)	65 (100)	spinal canal stenosis 12, demyelination 10, lumbar disc herniation 9, myelopathy 8, peripheral neuropathy 7, tumors 4, cervical spondylosis 2, other disease 13	NA	mean 20.7 months (1–168)	NA	Aminoff-Logue Score	NA	improved 59.5% stable 32.3% worsened 4.6%	improved 32.3% stable 46.7% worsened 4.4%
Jablawi F et al., 2020 [4]	40	mean 69.27, median 71 (53–84)	32 (80.0)	NA	NA		mean 20.2 months, median 10 months (1–120)	NA	NA	Aminoff-Logue Score	NA	improved 53% stable 28% worsened 20%	
Takai K et al., 2019 [1]	40	mean 67	33 (82.5)	29 (72.5)	31 (77.5)	lumbar spinal stenosis 14, no abnormality 5, spinal lesion of unknown etiology 3, prostatomegaly 3, intramedullary tumor 2, cervical spinal stenosis 1, multiple sclerosis 1, polyneuritis 1, spinal cord infarction 1	NA	NA	median 11 (misdiagnosis) vs. 4 months (no-misdiagnosis)	Aminoff-Logue Score	NA	NA	NA
Hunt R et al., 2018 [5]	37	median 62	28 (71.8)	26 (70.3)	22 (59.5)	ischaemia 4, Inflammatory 4, neoplasia 3, syrinx 3	NA	mean 22 (include arteriovenous aetiology in the differential diagnosis) vs. 281 days (exclude arteriovenous aetiology)	NA	NA	NA	NA	NA
Ortega-Suero G et al., 2017 [6]	7	mean 66.9 (50–80)	6 (85.7)	4 (57.1)	5 (71.4)	lumbar spinal stenosis 2, Restless legs syndrome 1, mixed headache 1, post-polio syndrome and syringomyelia 1	mean 31 months, median 24 months. mean 41.4 months (misdiagnosis) vs. 5 months (no-misdiagnosis)	NA	NA	Aminoff-Logue Score	improved 57% stable 14% worsened 29%	improved 57% stable 14% worsened 29%	improved 0% stable 86% worsened 14%
Barreras P et al., 2017 [7]	8	mean 63.9 (49–75)	7 (87.5)	4 (50.0)	8 (100)	transverse myelitis 7, neuromyelitis optica 1	NA	NA	NA	Aminoff-Logue Score	improved 85% stable 12.5% worsened 12.5%	NA	NA
Lee J et al., 2016 [8]	40	mean 58.2, median 58 (21–82)	27 (67.5)	24 (60.0)	7 (17.5)	myelitis 5, meningitis and arachnoiditis 1, spinal cord tumor 1	mean 10.15 months	NA	mean 12.16 months	Aminoff-Logue Score	improved 48.7% stable 35.8% worsened 15.3%	NA	NA
Brinjikji W et al., 2016 [9]	100	mean 65.0	43 of the misdiagnosed patients (81.1)	NA	53 (40.8)	spinal stenosis 13, myelopathy 10, transverse myelitis 9, ischemic myelopathy 4, peripheral neuropathy 3, myopathy 2, neuromyelitis optica 2, chronic inflammatory demyelinating polyneuropathy 2, Other 8	NA	mean 9.2 months, median 6 months	NA	Aminoff-Logue Score	NA	improved 32% stable 62% worsened 6%	NA
Zogopoulos P et al., 2016 [10]	14	mean 62.1 (42–74)	12 (71.4)	10 (71.4)	NA	NA	mean 13.5 months (1–36)	NA	NA	modified Rankin Scale	NA	improved 50% stable 50% worsened 0%	NA
Iovtchev I et al., 2015 [11]	7	mean 60.3 (30–72)	7 (100)	6 (85.7)	NA	spinal stenosis, myeloradiculitis, peripheral neuropathy and more.	mean 302.8 days (60–730)	NA	NA	Aminoff-Logue Score	NA	Aminoff-Logue Score of gait 5 n = 4, 4 n = 2, 3 n = 1	NA
Donghai W et al., 2013 [12]	326	mean 53.9	282 (86.5)	234 (71.8)	265 (81.3)	degenerative disc disease 120, myelitis 69, prostatic hyperplasia 17, spinal stenosis 13, intramedullary tumor 12, idiopathic scoliosis 9, syringomyelia 8, spinal cord injury 6, multiple sclerosis 5, tendinopathy 3, Guillan-Barre syndrome 3	mean 19.9 months	NA	NA	Aminoff-Logue Score	Improved 42.3% stable 53.1% worsened 4.5%	NA	NA
Cecchi PC et al., 2009 [13]	16	mean 58.4 (23–72)	14 (62.5)	12 (75.0)	NA	NA	NA	NA	mean 18.8 months (7–48)	Aminoff-Logue Score	improved 56.25% stable 37.5% worsened 6.255%	improved 50% stable 43.75% worsened 6.25%	improved 31.25% stable 62.5% worsened 6.25%
Aghakhani N et al., 2008 [14]	6 (paraplegic cases)	mean 53.3 (58–75)	4 (66.7)	3 (50.0)	NA	NA	NA	NA	mean 39 months (11–144)	American Spinal Injury Association Impairment Scale	NA	improved 100% stable 0 % worsened 0 %	NA
Jellema K et al., 2003 [15]	80	median 60.2 (34–79)	66 (82.5)	65 (85.0)	NA	NA	median 15 months (7 days–197 months)	NA	NA	NA	NA	NA	NA
Schick U et al., 2003 [16]	18	mean 60 (32–84)	16 (88.9)	10 (55.6)	7 (38.9)	tumor, polyneuropathy, Guillain-Barré syndrome, syringomyelia, and knee disease	mean 15 months (4–45)	NA	NA	MMT	NA	improved 38.9% stable 38.9 % worsened 22.2 %	NA
Atkinson JL et al., 2001 [17]	94	mean 63 (31–83)	75 (79.8)	54 (57.4)	NA	NA	mean 23 months (2–120)	NA	NA	Aminoff-Logue Score	NA	improved 98.9% stable 0% worsened 1.1%	NA
Huffmann BC et al., 1995 [18]	21	median58 (38–78)	19 (90.5)	14 (66.7)	9 (42.8)	tumor2, lumbar disc prolapse 2, prostate hypertrophy 1, polyneuropathy 1, funicular myelosis 1, knee disease 1	NA	NA	NA	Aminoff-Logue Score	NA	improved 95.2% stable 4.8% worsened 0%	NA

NA: Not applicable.

## Data Availability

The data used in this study are available from the corresponding author upon request.
